# Chemical Profiling by High‐Performance Liquid Chromatography With Diode Array Detection and Gas Chromatography‐Mass Spectrometry Analysis and Antimicrobial Potential of *Achillea santolina* Plant Extracts against Extended‐Spectrum Beta‐Lactamase‐producing *Escherichia coli* and Fungal Pathogens

**DOI:** 10.1002/cbdv.202403064

**Published:** 2025-01-20

**Authors:** Chahna Renda, Sadou Nina, Bouzana Amina, Bougouizi Amina, Haouame Imane, Rebbas Khellaf, Stefania Garzoli, Hamdi Bendif

**Affiliations:** ^1^ Department of Biology Laboratory of Plant Biology and Environment “Medicinal plants” Axis Faculty of Sciences_ Badji Mokhtar University Annaba Algeria; ^2^ Department of Nature and Life Sciences, Laboratory of Interactions, Biodiversity, Ecosystems and Biotechnology, Faculty of Sciences University 20 August 1955 Skikda Algeria; ^3^ Department of Natural and Life Sciences Faculty of Sciences University of M'sila M'sila Algeria; ^4^ Department of Chemistry and Technologies of Drug Sapienza University Rome Italy; ^5^ Biology Department, College of Science Imam Mohammad Ibn Saud Islamic University (IMSIU) Riyadh Saudi Arabia

**Keywords:** *Achillea santolina*, antibacterial activity, antifungal activity, HPLC‐DAD, GC‐MS

## Abstract

The study examines the chemical composition and antimicrobial properties of petroleum ether and hydro‐methanolic extracts of *Achillea santolina* from Algeria. Chemical profiling was performed using high‐performance liquid chromatography with diode array detection for the hydro‐methanolic extract and gas chromatography‐mass spectrometry for the petroleum ether extract. Antibacterial and antifungal activities were evaluated using the disc diffusion method and broth dilution technique. Epicatechin (24.54 mg/g extract), and camphor (19.18%) were identified as main compounds in the hydro‐methanolic and petroleum ether extracts, respectively. Both extracts showed significant antibacterial effects against extended‐spectrum beta‐lactamase‐producing *Escherichia coli* strains, with inhibition diameters ranging from 10 to 13 mm, and minimum inhibitory concentration (MIC) values between 0.78 and 3.5 mg/mL. Anti‐fungal activity was also notable, particularly against *Candida albicans*, with an inhibition diameter of 14 mm, and MIC values between 0.39 and 1.56 mg/mL. The hydro‐methanolic extract showed up to 90% of growth inhibition against *Aspergillus niger*. These findings suggest that *A. santolina* could serve as a promising source of antimicrobial compounds to combat resistant pathogens.

## Graphical abstract

Investigation of the chemical profile and antimicrobial power of *Achillea Santolina* extracts

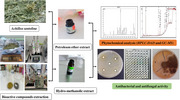

## 1 Introduction

Long before the discovery of microbes, certain plants were recognized for their healing properties, now understood as antimicrobial compounds. Traditionally used for treating infections, many of these plants remain in use and are actively studied for their effectiveness against various pathogens [1].

The global rise in extended‐spectrum beta‐lactamase (ESBL)‐producing *Escherichia coli* strains, in African countries like Algeria, poses a significant problem due to their resistance to certain antibiotics, specifically in urinary tract infections [2]. *E. coli*, a Gram‐negative facultative anaerobe bacterium, is a major contributor to various infections and is associated with a wide range of gastrointestinal diseases, including conditions such as enteritis, bacteremia, diarrhea, and urinary tract infections [3]. The excessive use of antibiotics has significantly contributed to the growing drug resistance of *E. coli*, posing a serious threat to public health. Consequently, many researchers are exploring natural compounds from plant extracts as alternative approaches to combat ESBL‐producing *E. coli* [3].

Additionally, fungal infections are increasing, especially *candidiasis*, with *Candida albicans* as the most prevalent symbiotic fungus, commonly coexisting with humans and animals as a part of the normal microbial flora [4]. Infections caused by *C. albicans* typically arise when the immune system is weakened, leading to conditions such as oral, vaginal, skin, and nail candidiasis, as well as more severe systemic fungal infections [5]. If left untreated, *C. albicans* infections can progress to bloodstream infections, causing symptoms such as fever, weakness, appetite loss, anemia, and organ failure, posing a significant threat to the health and survival of affected individuals [6]. In recent years, several antibiotics have been used to combat infections caused by *C. albicans*, providing a broader range of antifungal activity effective against complex *C. albicans* infections. However, the use of antibiotics poses a number of challenges [7]. Similarly, the fungal growth of molds like *Aspergillus* spp., including *Aspergillus niger*, is an important cause of food spoilage and intoxication, with increasing resistance to standard treatments [9]. Therefore, there is a growing need for new, environmentally friendly methods that can either replace or reduce the reliance on antibiotics.

The growing demand for alternative treatments with herbal medicine has grown in recent years, renewing interest in plants that are safe, culturally acceptable, and effective [10]. *Achillea santolina*, our plant of interest, belongs to the genus *Achillea* and family *Asteraceae*, which includes approximately 115 species primarily found across North Africa, Europe, and Asia [11]. In Algeria, around five species of *Achillea*, including *A. santolina*, are widely distributed [12]. This plant has a longstanding role in traditional medicine for treating various ailments [13]. Many *Achillea* species are known for their antiseptic and infection‐fighting properties [14].

The bioactive properties of *Achillea* essential oils and extracts, such as analgesic, antioxidant, anti‐inflammatory, and antimicrobial activities [15], have been well documented.

To the best of our knowledge, no studies have explored the chemical composition or evaluated the antimicrobial activity of *A. santolina* extracts in Algeria, while research on its essential oils has been conducted in regions such as Egypt [16], Jordan [17], and Iran [18]. This study is the first to evaluate the antimicrobial properties of *A. santolina* extracts, focusing on their effectiveness against resistant bacteria, particularly those producing ESBL. This work aims to explore the chemical composition of *A. santolina* extracts using gas chromatography‐mass spectrometry (GC‐MS) and high‐performance liquid chromatography with diode array detection (HPLC‐DAD) and to evaluate their anti‐bacterial and anti‐fungal activities, with a particular focus on their potential against resistant *E. coli* producing (ESBL) and other antifungal pathogens.

## 2 Results and Discussion

### 2.1 Chemical Composition by GC‐MS Analysis of the Petroleum Ether Extract of *A. santolina*

**FIGURE 1:**
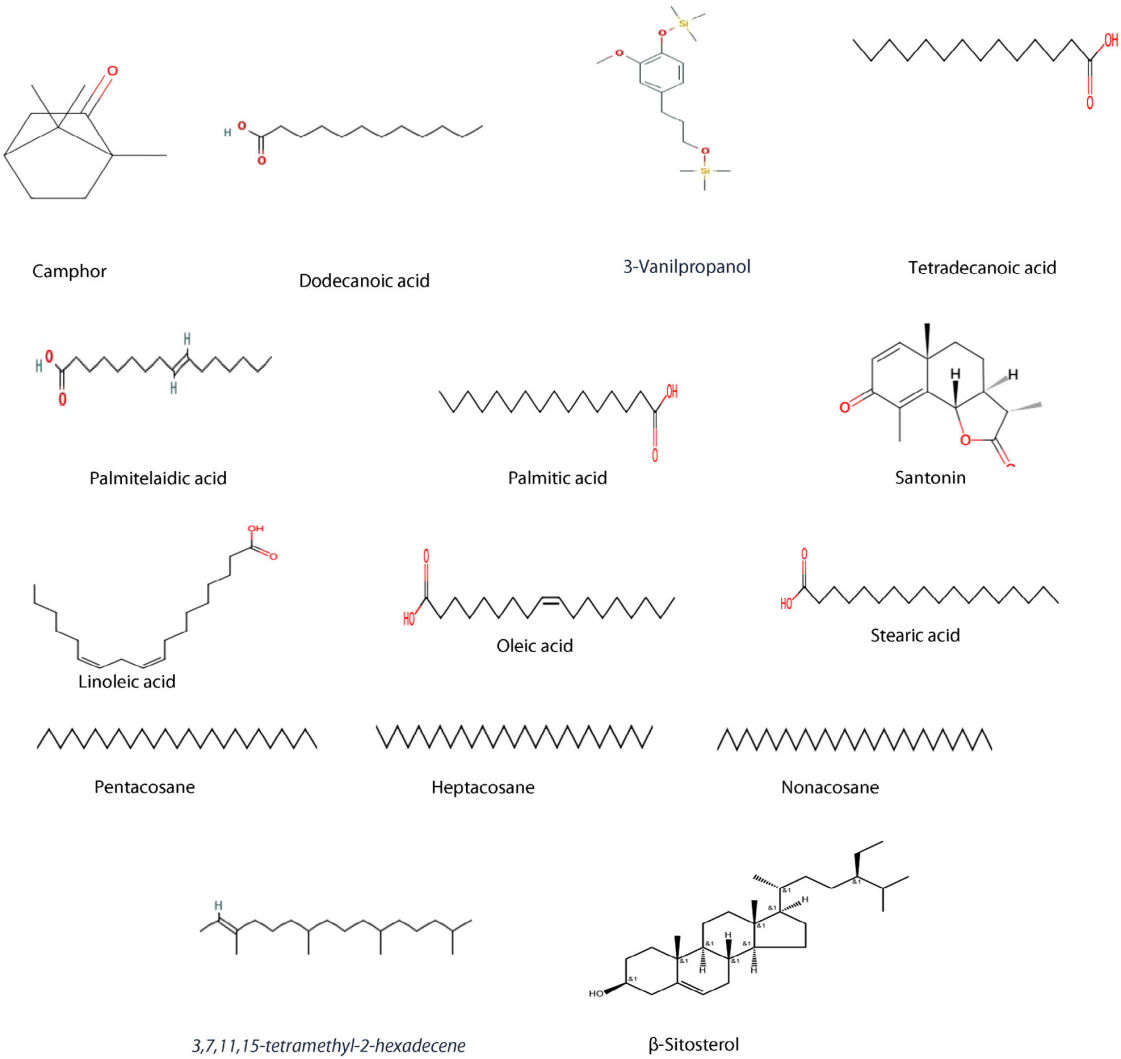
Chemical structures of identified compounds in the petroleum ether extract of *A. santolina*.

**FIGURE 2:**
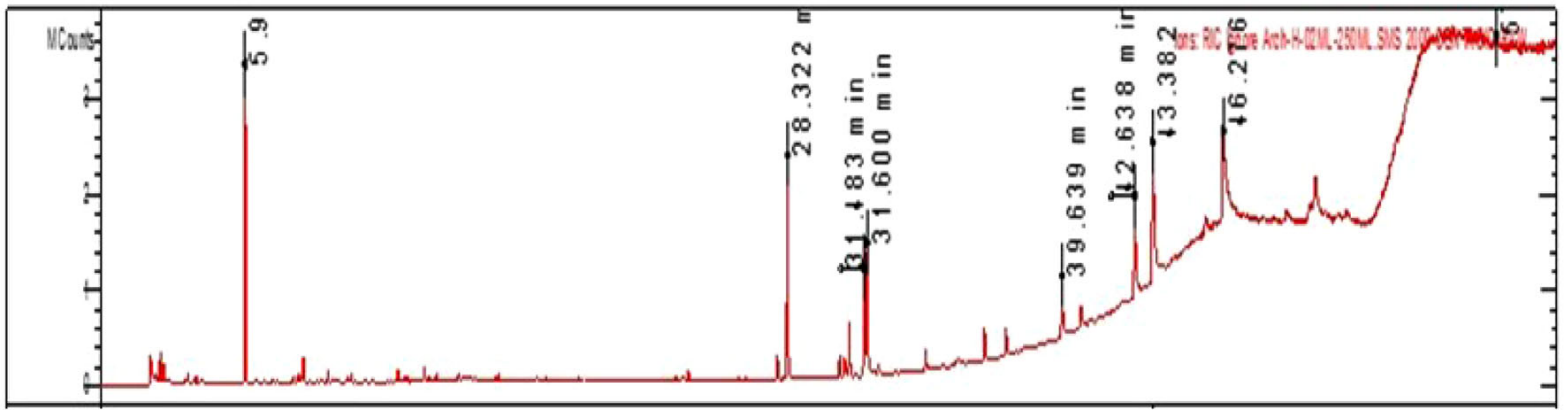
Gas chromatography‐mass spectrometry (GC‐MS) chromatogram of *A. santolina* petroleum ether extract.

The GC‐MS analysis of the petroleum ether extract of *A. santolina* has identified 15 compounds, as shown in Table 1 and Figure 1. The primary constituents included camphor (19.2 %), palmitic acid (15.2 %), oleic acid (11.5 %), nonacosane (10.6 %), linoleic acid (7.7 %), and heptacosane (5.2 %). Camphor is commonly identified as a major compound in various *Achillea* species [19]. The GC/MS chromatogram is reported in Figure 2. The notable high camphor content in *A. santolina*, compared to other *Achillea* species, suggests that it belongs to a camphor‐dominant chemotype. The other constituents found in significant amounts were: dodecanoic acid, 3‐vanilpropanol, tetradecanoic acid, palmitelaidic acid, santonine, 3,7,11,15‐tetramethyl‐2‐hexadecene, stearic acid, pentacosane, and β‐sitosterol. The presence of these compounds or their interaction with other secondary metabolites may enhance the targeted elimination of microbial strains.

**TABLE 1:** Chemical composition of the petroleum ether extract of *A. santolina* from the Aflou region (Algeria) using the gas chromatography‐mass spectrometry (GC‐MS) technique.

Peak No.	RT	Phenolic compounds	Molecular formula	RI	Petroleum ether extract (%)
1	5.964	Camphor	C₁₀H₁₆O	1144	19.2
2	19.766	Dodecanoic acid	C₁₂H₂₄O₂	1657	tr
3	23.74	3‐Vanilpropanol	C_16_H_30_O_3_Si_2_	1813	tr
4	24.225	Tetradecanoic acid	C₁₄H₂₈O₂	1850	tr
5	27.894	Palmitelaidic acid	C₁₆H₃₀O₂	2025	tr
6	28.322	Palmitic acid	C₁₆H₃₂O₂	2045	15.2
7	30661	Santonine	C_15_H_18_O_3_	2172	tr
9	30.886	3,7,11,15‐tetramethyl‐2‐hexadecene	C₂₀H₄₀	2180	tr
10	31.483	Linoleic acid	C₁₈H₃₂O₂	2200	7.7
11	31.6	Oleic acid	C₁₈H₃₄O₂	2208	11.5
12	32.091	Stearic acid	C₁₈H₃₆O₂	2240	tr
13	36.59	Pentacosane	C₂₅H₅₂	2500	tr
14	39.639	Heptacosane	C₂₇H₅₆	2700	5.2
15	42.638	Nonacosane	C₂₉H₆₀	2900	10.6
16	50.171	β‐Sitosterol	C₂₉H₅₀O	3380	tr

Abbreviations: RI, retention index; Rt, retention time; tr, percentage values ˂0.1%.

**FIGURE 3:**
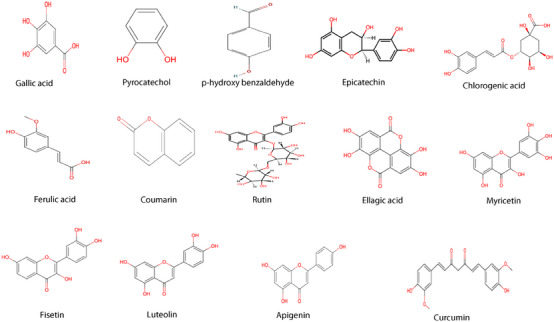
Chemical structures of identified compounds in the hydro‐methanolic extract of *A. santolina*.

**FIGURE 4:**
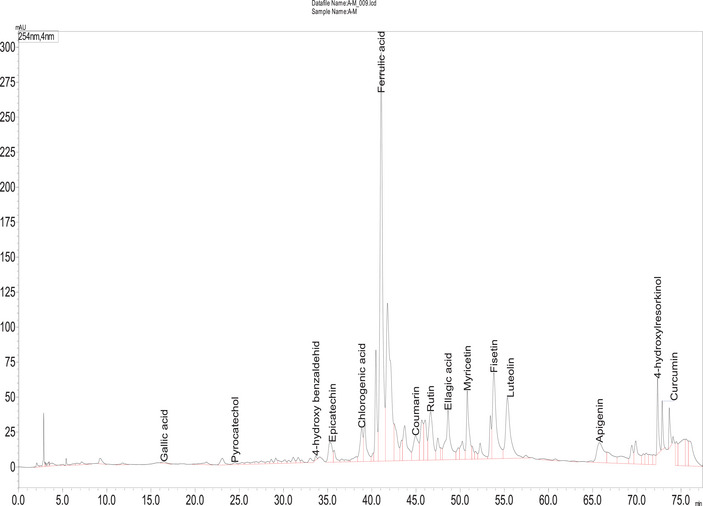
High‐performance liquid chromatography with diode array detection (HPLC‐DAD) chromatogram of *A. santolina* at 254 nm (Inertsil ODS‐3 column [4 µm, 4 × 150 mm], mobile phase 0.1% acetic acid‐methanol [gradient elution], flow rate 1 ml/min, and diode array detection 254 nm).

Berramdane et al. [20] reported that *A. santolina* essential oils from Algeria primarily contain camphor and 1,8‐cineole (see Figures 1, 2, 3, 4). In another Algerian region, *A. santolina* oils had cis‐sabinene hydrate and 1,8‐cineole as major components [21], while *A. santolina* grown in Egypt contains: 1,8‐cineole, camphor, bomeol, pinenes, artemisia ketone, and santolina alcohol as main compounds [22].

### 2.2 Quantitative Analysis of Phenolic Compounds by HPLC‐DAD

A reverse‐phase HPLC‐DAD method was used to analyze the phenolic compounds. For an optimal separation, various solvent combinations and flow rates were tested to acheive optimal separation. All chemicals used in this study were of HPLC grade. Compounds were identified by comparing their spectroscopic properties and retention times with reference standards (Table 2 and Figure 3). HPLC‐DAD chromatogram was reported in Figure 4.

**TABLE 2:** Phenolic composition of the extracts of the hydro‐methanol extract of *A. santolina* by high‐performance liquid chromatography with diode array detection (HPLC‐DAD) (mg/g extract).

Peak No.	Phenolic compounds	Molecular Formula	RT	Calib. eq.	R^2^	Hydro‐methanolic extract (mg/g extract)
1	Gallic acid	C₇H₆O₅	15.225	*y* = 45540*x* ‐84708	0.995	0.31
2	Pyrocatechol	C₆H₆O₂	24.625	*y* = 3772.8*x* + 23692	0.9999	0.32
3	p‐Hydroxy benzaldehyde	C₇H₆O₃	33.367	*y* = 34376*x* + 4239.6	0.9996	0.10
4	Epicatechin	C₁₅H₁₄O₆	35.278	*y* = 2097.6x + 7998.2	0.998	24.54
5	Chlorogenic acid	C₁₆H₁₈O_9_	40.094	*y* = 46920*x* ‐ 36953	0.9995	1.55
6	Ferulic acid	C₁₀H₁₀O₄	42.564	*y* = 42245*x* + 110701	0.9992	17.56
7	Coumarin	C₉H₆O₂	45.178	y = 81802x + 153471	0.9968	0.80
8	Rutin	C₂₇H₃₀O₁₆	47.527	*y* = 47899*x* + 56096	0.9997	2.77
9	Ellagic acid	C₁₄H₆O₈	50.005	*y* = 235073*x* ‐ 7E+06	0.9808	4.37
10	Myricetin	C₁₅H₁₀O_8_	50.368	*y* = 136859*x* + 71185	0.995	0.83
11	Fisetin	C₁₅H₁₀O₆	51.243	*y* = 100784*x* + 16688	0.9984	2.31
12	Luteolin	C₁₅H₁₀O₅	57.872	*y* = 89569*x* ‐ 62198	0.9995	2.38
13	Apigenin	C₁₅H₁₀O₅	64.071	*y* = 71990*x* + 62472	0.9996	1.24
14	Curcumin	C₂₁H₂₀O₆	72.622	*y* = 15850*x* + 140009	0.9932	1.44

Abbreviations: RT, retention time; R^2^, regression coefficient.

Fourteen compounds were detected in *A. santolina*, among which epicatechin was the predominant (24.54 mg/g extract), followed by ferulic acid (17.56 mg/g), ellagic acid (4.37 mg/g), rutin (2.77 mg/g), luteolin (2.38 mg/g), and fisetin (2.31 mg/g). Other constituents were chlorogenic acid (1.55 mg/g), curcumin (1.44 mg/g), apigenin (1.24 mg/g), myricetin (0.83 mg/g), coumarin (0.80 mg/g), pyrocatechol, gallic acid, and p‐hydroxy benzaldehyde in trace amounts. Chlorogenic acid, rutin, and apigenin are found in *Achillea millefolium* L. s.l. [23]. Epicatechin, a flavonoid commonly found in fruits and green tea, is obtained as a pale yellow powder and exhibits antibacterial activity by disrupting bacterial membranes and inhibiting toxin production [24]. Ferulic acid, typically isolated as a white crystalline powder, enhances the efficacy of quinolone antibiotics [25]. Plant‐derived compounds like ferulic, gallic acid (a white crystalline powder), and luteolin (a yellow crystalline powder) exhibit antimicrobial effects, disrupting membrane integrity [26]. Chlorogenic acid, widely present in foods, and obtained as a greenish‐brown crystalline powder, disrupts microbial cell membranes and metabolism, leading to cell death [27]. Gallic acid, a major polyphenol, combats viral and bacterial infections [28].

### 2.3 Antimicrobial Activity

The rise of drug‐resistant microbes like *C. albicans*, *A. niger*, and *E. coli* poses a real public health challenge, particularly due to the limited effectiveness of current antibiotics and antifungal treatments. *E. coli* is a leading cause of urinary tract infections and produces ESBL, while *A. niger* and *C. albicans* are involved in serious opportunistic infections [29]. This underscores the urgent need for new therapeutic agents with novel mechanisms of action.

The findings of the antimicrobial activity revealed that both hydro‐methanol and petroleum ether extracts of *A. santolina* exhibited significant and variable efficacy against different microbial strains, including *E. coli*, *C. albicans*, and *A. niger* (Table 3).

**TABLE 3:** Antimicrobial efficacy of *A. santolina* petroleum ether and hydro‐methanolic extracts.

Extracts Microbial strains	Petroleum ether extract		Hydro‐methanolic extract	
	IZ (mm)	MIC (mg/mL)	IZ (mm)	MIC (mg/mL)
*E. coli* ATCC 25922	R	R	10 ± 0.94^a^	1.56
*E. coli* ATCC 25933	13 ± 0.12^a^	3.5	R	R
*E. coli* 01	R	R	12 ± 0.54^b^	1.56
*E. coli* 02	13 ± 0.20^a^	1.56	10 ± 0.61^a^	1.56
*E. coli* 03	R	R	11 ± 0.37^c^	1.56
*E. coli* 04	13 ± 1.02^a^	0.78	R	R
*C. albicans* ATCC 10231	10 ± 0.63^b^	1.56	R	R
*C. albicans* 01	R	R	R	R
*C. albicans* 02	10 ± 0.22^b^	0.39	10 ± 1.52^a^	1.56

Growth inhibition percentage (%)				
	Concentration (mg/mL)	% Inhibition	Concentration (mg/mL)	% Inhibition
*A. niger* 01	25	75°°°	50	90°°°
*A. niger* 02	25	5°	NA	NA
*A. niger* 03	12,5	60°°	NA	NA

*Note*: R: Resistant; NA: No activity; ° :30 to 40%: low activity; °°: 50 to 60%: moderate activity; °°°: >70%: excellent activity. The values in the columns, indicated by unrelated characters (a, b, c), have differences significant (*p* < 0.05).

The petroleum ether extract demonstrated higher antibacterial activity against several strains of *E. coli*, particularly *E. coli ATCC 25933*, and two ESBL‐producing strains *E. coli* 04 and *E. coli* 02, with inhibition diameters of 13 ± 0.20 mm and MIC ranging from 0.78 to 3.5 mg/mL. In comparison, the hydro‐methanol extract also showed notable antibacterial activity, especially against *E. coli ATCC 25922, E. coli 01, E. coli 03, and E. coli 02*, with inhibition diameters ranging from 10 ± 0.61 to 12 ± 0.54 mm and MIC of 1.56 mg/mL. Our findings are consistent with those of Sharifi‐rad [30], who reported that the essential oil of *Achillea wilhelmsii*, showed a substantial antibacterial effect against ESBL‐producing *E. coli* isolates, with MIC ranging from 0.5 to 4 mg/mL [30].

These findings were compared with those from studies on other *Achillea* species and revealed that the antibacterial activity of *A. santolina* was lower than that observed in *Achillea* species from the Egyptian Sahara [31]. Similar results from Jordan showed that *A. santolina* essential oils demonstrated moderate antibacterial activity on *E. coli*, with inhibition zones ranging from 6.0 to 16.5 mm and MIC and minimum bactericidal concentration values between 60.0 and 480.0 µg/mL [32]. Such variations are due to the difference in chemical composition and compound abundance, which can be influenced by environmental factors, including location, season, climate, plant genetics, and extraction methods [33]. Therefore, the antibacterial characteristics of *A. santolina* are likely related to their high concentrations of 1, 8‐cineol, and camphor previously identified in the petroleum ether extract [20].

Regarding yeasts, *A. santolina* extracts were effective, particularly the petroleum ether extract, which inhibited *C. albicans ATCC 10231* and *C. albicans* 02, with inhibition zones of 10 ± 0.63 and 10 ± 0.22 mm and MICs of 1.56 and 0.39 mg/mL, respectively. The hydro‐methanolic extract also showed antifungal potential against *C. albicans* 02 with an inhibition zone of 10 ± 1.52 mm. Given *C. albicans*' association with various infections [4], *A. santolina* extracts could offer a natural antifungal treatment.

For molds, both extracts displayed antifungal activity against *A. niger*. The petroleum ether extract achieved moderate inhibition of 75% and 60% for *A. niger* 01 and *A. niger* 03, while the hydro‐methanolic extract showed higher inhibition, reaching 90% against *A. niger* 01, although both *A. niger* 02 and *A. niger* 03 were resistant to it. The antimicrobial efficacy of *A. santolina* extracts can be attributed to their rich chemical composition (Tables 1 and 2).

HPLC analysis showed that *A. santolina* contains significant amounts of epicatechin, chlorogenic acid, gallic acid, and rutin, which likely contribute to its antibacterial properties. Epicatechin, found in plants like *Camellia sinensis*, has demonstrated antibacterial activity [34]. Similarly, gallic acid and chlorogenic acid are known for their antibacterial effects in plants such as *Achillea millefolium* and burdock leaves [28]. The interaction of phenolic and flavonoid compounds with cell membrane proteins may explain varying antibacterial activity among *Achillea* species [35].

GC‐MS analysis of the Petroleum ether extract of *A. santolina* showed identified several major compounds including camphor, palmitic acid, oleic acid, nonacosane, linoleic acid, heptacosane, and stearic acid. Studies on various Achillea species have demonstrated camphor's strong antimicrobial activity [20], particularly against *C. albicans* [15]. Additionally, the antimicrobial properties of palmitic acid, heptacosane, and nonacosane have been well‐documented in the literature [36]. The presence of oleic and linoleic acids further contributes to the antibacterial effects of A. santolina, suggesting that these minor compounds may act synergistically with the primary bioactive constituents, thereby enhancing the overall therapeutic potential of the plant [37].

The differences in results may be attributed to variations in chemical composition and the abundance of compounds, which are influenced by several environmental factors such as location, season, climate, plant genetics, and extraction methods [33]. Additionally, the type of extract used can significantly affect the antimicrobial properties [11], as previous research has predominantly focused on essential oils, whereas our study focused on hydro‐methanolic and petroleum ether extracts. The observed antimicrobial activity could be attributed to the higher concentrations of flavonoids and phenolic compounds in the *Achillea* species examined [18]. Flavonoids, as noted by Salami, inhibit microbial growth through various mechanisms, including disruption of nucleic acid synthesis, plasma membrane function, and interference with energy metabolism [35]. The antimicrobial activity against the tested microorganisms could also result from synergistic interactions among the major compounds present in the extracts [37]. Given its effectiveness against a wide range of infections, *A. santolina* extracts may offer a promising natural treatment for both bacterial and fungal infections, emphasizing the potential of plant‐based alternatives in combating microbial diseases.

## 3 Conclusions

This study demonstrates the significant antimicrobial potential of *A. santolina* from Algeria, which may be attributed to the presence of several key compounds. In the hydro‐methanolic extract, epicatechin (24.5 mg/g) and ferulic acid (17.56 mg/g) were found in notable concentrations, while the petroleum ether extract contains compounds such as camphor (19.2%), palmitic acid (15.2%), oleic acid (11.5%), and nonacosane (10.6%). Although the exact compound responsible for the antimicrobial activity remains unclear, these molecules collectively contribute to the plant's efficacy against *C. albicans* and *A. niger*, as well as its significant antibacterial effect against ESBL‐producing *E. coli* isolates.

These findings support further exploration of *A. santolina* as a natural source for developing antimicrobial agents, especially for combating resistant pathogens. The study provides valuable insights into the chemical composition and antimicrobial properties of *A. santolina*, which could lead future research to the development of novel antimicrobial treatments. Future studies should focus on isolating and testing the active compounds in *A. santolina* to further evaluate their efficacy against a wider range of pathogens. Additionally, in vivo tests and clinical trials are important to assess the therapeutic potential of these compounds in real‐world applications. This research aims to develop plant‐based antimicrobial agents that could improve existing treatments and provide effective solutions to the growing issue of antimicrobial resistance.

## 4 Experimental

### 4.1 Plant Material

The aerial parts of *A. santolina* were collected in June 2021 from Aflou, Algeria, identified by Pr. K. Rebbas (voucher N° KR0030), washed, air‐dried, and powdered. Fifty grams were extracted by maceration with petroleum ether and 80% methanol for 24 h, then filtered, evaporated, and stored at 4°C for analysis.

### 4.2 HPLC Analyses (HPLC‐DAD)

The phenolic profile of the hydro‐methanolic extract of *A. santolina* was analyzed using a Shimadzu HPLC‐DAD (Shimadzu Corporation, Kyoto, Japan). The system included a solvent delivery unit (LC‐20AT) and a diode array detector (SPD‐M20A), the analysis was performed based on 27 standards [38] and controlled by LC‐solution software (CBM‐20A System Controller Shimadzu). Chromatographic separation was conducted on an Inertsil ODS‐3 column at 40°C. The sample (8 mg/mL in 80% methanol/water) was filtered and injected (20 µL).

The mobile phase consisted of 0.1% acetic acid in water (A) and methanol (B), with a 40‐minute gradient at a 1.5 mL/min flow rate. Detection occurred at 230–350 nm, with phenolic compounds identified by retention times and ultraviolet data, expressed as mg/g of dry extract.

### 4.3 Fatty Acids Analysis by GC–MS

In a 25 mL flask, 25 mg of *A. santolina* petroleum ether extract was combined with 2 mL of 0.5 N NaOH, sealed, and heated in a boiling water bath for 5 min. After cooling, 2 mL of BF₃–MeOH was added, and the mixture was reheated at 80°C for 3 min. Once cooled, 5 mL of saturated NaCl solution was added, followed by shaking and two *n*‐hexane extractions (20 mL each) [38]. For analysis, a Shimadzu GC17 AAF gas chromatograph with a DB‐1 non‐polar capillary column was used. Helium served as the carrier gas (1.2–1.3 mL/min), with injector and detector temperatures set at 250 and 270°C. The oven temperature started at 100°C for 5 min, ramped to 238°C at 3°C/min, and held for 9 min.

Fatty acid methyl esters (FAMEs) were analyzed by GC‐flame ionization detector and quantified via GC Solution software. GC‐MS analysis utilized a Varian Saturn 2100T ion trap analyzer at 70 eV, with detector, injector, and transfer line temperatures at 240, 220, and 290°C, respectively, identifying FAMEs with NIST/Wiley library matches and Supelco 37 standards [39].

### 4.4 Tested Microorganisms

The antimicrobial efficacy of the extracts was tested on four ESBL‐producing *E. coli* strains, five fungal strains (two *C. albicans* and three *A. niger*), and three reference strains from the Pasteur Institute in Algiers (*E. coli* ATCC 25922, *E. coli ATCC 25933*, and *C. albicans ATCC 10231*).

### 4.5 Antimicrobial Efficacy of *A. santolina* Extracts

#### 4.5.1 Isolation and Characterization of ESBL‐producing *E. coli* and Fungal Strains

*E. coli* strains linked to urinary tract infections were obtained from a Skikda laboratory, representing patients of various ages: a newborn, a 5‐, a 35‐, and an 81‐year‐old. Three isolates were from females and one from a male. Strain identification and antibiograms were conducted using the Vitek 2 compact system. ESBL production was confirmed via double‐disc synergy and polymerase chain reaction (PCR) detection of the blaCTX‐M gene in all strains using standard PCR. The primer sequences and protocol were previously described [40]. All four multidrug‐resistant isolates resisted at least three antibiotic classes, including ampicillin, first‐ and third‐generation cephalosporins, ciprofloxacin, trimethoprim, and sulfamethoxazole; two also resisted amoxicillin/clavulanic acid.

#### 4.5.2 Fungal Strains Isolation

Fungal strains were obtained from a private laboratory in Skikda and were isolated from various samples, including vaginal swabs, between toes, tongue, and nails. These samples were collected from two female and three male patients, aged between 30 and 60 years. The identification was conducted using both macroscopic and microscopic examinations, along with the germ tube test as described by Mackenzie [41], to confirm the presence of *C. albicans*.

#### 4.5.3 Evaluation of the Antimicrobial Activity

Antimicrobial activity against *E. coli* (ESBL) and *C. albicans* was evaluated using the Kirby‐Bauer disk diffusion method on Mueller‐Hinton agar [42], and MIC was determined by broth microdilution (CLSI, 2018), with interpretation according to Ponce et al. *A. niger* growth inhibition was evaluated via disk diffusion on chloramphenicol‐supplemented Sabouraud agar, calculated using Hajji [43] formula:

%Inhibitiongrowth=(dc−dt)/dc×100

where *dc* represents the colony diameter in the control dish, and *dt* is the diameter in the treatment dish. Results were interpreted according to the scale proposed by Abd‐Ellatif et al. [44].

### 4.6 Statistical Analysis

The in vitro results were presented as mean ± SD of three measurements and the obtained data were evaluated using Tukey's test and one‐way analysis of variance, using SPSS software. The differences are regarded as significant when *p*‐values < 0.05.

## Author Contributions

**Chahna Renda**: data curation, formal analysis, writing original draft. **Sadou Nina**: data curation, writing–review and editing. **Bouzana Amina**: Investigation, writing–review and editing. **Bougouizi Amina**: writing–review and editing. **Haouame Imane**: data curation, formal analysis. **Bendif Hamdi**: writing–review and editing, validation, investigation. **Stefania Garzoli**: writing–review and editing and supervision. **Rebbas Khellaf**: resources.

## Conflicts of Interest

The authors declare no conflicts of interest.

## Data Availability

Data will be made available on request.
